# EPR Spectroscopy of a Clinically Active (1:2) Copper(II)-Histidine Complex Used in the Treatment of Menkes Disease: A Fourier Transform Analysis of a Fluid CW-EPR Spectrum

**DOI:** 10.3390/molecules19010980

**Published:** 2014-01-15

**Authors:** Lukas Gala, Michael Lawson, Klaudia Jomova, Lubomir Zelenicky, Andrea Congradyova, Milan Mazur, Marian Valko

**Affiliations:** 1 Faculty of Chemical and Food Technology, Slovak Technical University, Bratislava SK-812 37, Slovakia; E-Mails: lukas.gala19@gmail.com (L.G.); alpinenewt@gmail.com (M.L.); milan.mazur@stuba.sk (M.M.); 2 Faculty of Natural Sciences, Constantine The Philosopher University, Nitra SK-949 74, Slovakia; E-Mails: kjomova@ukf.sk (K.J.); lzelenicky@ukf.sk (L.Z.); andrea.congradyova@ukf.sk (A.C.)

**Keywords:** copper-histidine complex, copper metabolism, Menkes disease, EPR spectroscopy, FT-EPR spectroscopy

## Abstract

Redox active transition metal ions (e.g., iron and copper) have been implicated in the etiology of many oxidative stress-related diseases including also neurodegenerative disorders. Unbound copper can catalyze formation of reactive oxygen species (hydroxyl radicals) via Fenton reaction/Haber–Weiss chemistry and therefore, under physiological conditions, free copper is potentially toxic and very rarely exists inside cells. Copper(II) bound to the aminoacid l-histidine represents a species discovered in blood in the mid 60s and since then extensive research on this complex was carried out. Copper bound to l-histidine represents an exchangeable pool of copper(II) in equilibrium with the most abundant blood plasma protein, human serum albumin. The structure of this complex, in aqueous solution, has been a subject of many studies and reviews, however without convincing success. The significance of the (1:2) copper(II)-l-histidine complex at physiological pH documents its therapeutic applications in the treatment of Menkes disease and more recently in the treatment of infantile hypertrophic cardioencephalomyopathy. While recently the (1:2) Cu(II)-l-His complex has been successfully crystallized and the crystal structure was solved by X-ray diffraction, the structure of the complex in fluid solution at physiological pH is not satisfactorily known. The aim of this paper is to study the (1:2) Cu(II)-l-histidine complex at low temperatures by X-band and S-band EPR spectroscopy and at physiological pH at room temperature by Fourier transform CW-EPR spectroscopy.

## 1. Introduction

Copper is an essential metal element necessary for all living organisms [1]. It is an integral part of many copper-containing enzymes involved in various biological processes, such as photosynthesis, respiration, redox-metal metabolism, neurological functions and other processes.

The balance between copper needs and toxicity is tightly regulated at the cellular level and at the tissue and organ levels [2]. Free (unbound) copper in excess of physiological requirements is toxic, since it is known to catalyse formation of free radicals (mainly via the Fenton reaction) which in turn may lead to oxidation of important biological molecules including DNA, proteins, as well as biological membranes [3]. Therefore copper is present in biological systems as an integral part of enzymes or it is transported by proteins such as serum albumins. Also, chelation of copper by biologically active small molecular weight molecules has many physiological functions and in addition to this, copper chelation reduces its capacity to catalyse formation of free radicals [4].

**Figure 1:**
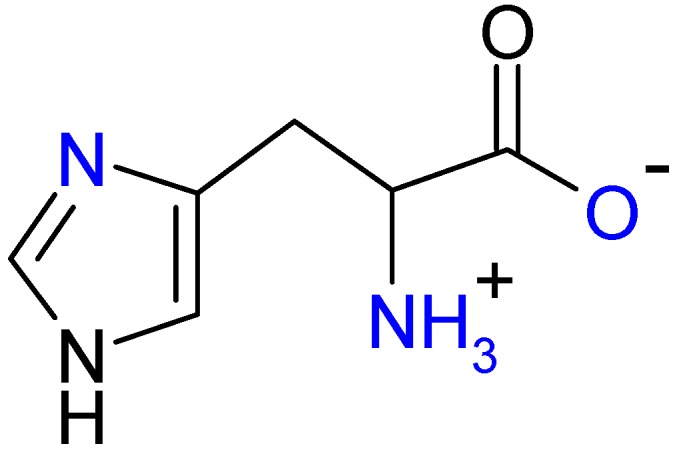
A potential tridentate ligand, l-histidine (HL).

One of the very important copper chelators is the amino acid l-histidine, which plays an important role in copper transport prior to its entry into cellular transport systems and incorporation into enzymes and proteins (Figure 1) [5]. Copper(II) bound to l-histidine is in equilibrium with human serum albumin and may undergo mutual exchange which modulates the bioavailability of copper to the cell.

The breakdown of copper metabolism results in various disease states of an organism [6]. Menkes disease is a genetic disease usually affecting male infants, characterized by a disorder of copper transport which affects levels of copper in the body with clinical manifestation of copper deficiency [7]. Clinical diagnosis of Menkes Disease is often difficult due to the fact that affected newborns usually appear healthy at birth, with almost no symptoms for the first 2–3 months. Currently there is no effective treatment for Menkes diseases, however copper treatment in the form of copper salts (copper chloride, copper sulfate, copper acetate) or copper histidine are available. The medicinal potential of copper-histidine complex has resulted in the increased interest of scientists in solving its structure. The exact role of l-histidine in copper-albumin interaction (albumin is a copper carrier) in cellular uptake of copper as well as in Menkes disease is not well understood. The exact characterization of copper-l-histidine complex in aqueous environment has been attempted, however without a detailed structural description [8,9,10,11,12,13,14,15,16].

The problems associated with the structural characterization of copper(II)-histidine species in solution are caused by the variability of l-histidine in coordinating metal ions. l-Histidine has three potential metal-binding sites and can bind to metals as mono-, bi- and tri-dentate ligands [5]. Moreover, its binding mode depends on the pH of the solution.

The only success with respect to direct structural elucidation of Cu(II)-l-histidine complex has been achieved in solid state by means of X-ray diffraction studies. The X-ray results have shown that the (1:2) copper(II)-l-histidine complex in the solid state is five-coordinate, possessing distorted square-pyramidal geometry with bidentate and tridentate l-histidine ligands [17].

EPR spectroscopy is a very sensitive technique for the elucidation of the structure of paramagnetic species in solution [18]. A unique feature of EPR is its ability to elucidate the directly bonded magnetically active atoms to a paramagnetic center of the complex and to describe the stereochemistry around a metal ion. This is substantiated by the resolution of superhyperfine (shf) structure in EPR spectra due to the magnetically active nuclei of donor atoms in metal complexes which provide valuable information about the nature of metal-ligand bonding [19].

In order to characterize the exact nature of directly bonded ligand atoms to copper as well as the stereochemistry around copper ion in aqueous solution, we present here the results of EPR study of (1:2) copper(II)-l-histidine complex. We believe that the detailed structural characterization of copper-l-histidine species in a fluid environment has biological importance and significance in view of a better understanding of the mechanism of action of the therapeutically active copper(II) complex.

## 2. Results and Discussion

### 2.1. EPR Theory

In our approach we assume that second order dynamical shifts are negligible and that the linewidth is independent of the magnetic field. Under these assumptions the line pattern generated by copper hyperfine interaction (I = 3/2) is given by [20,21]: 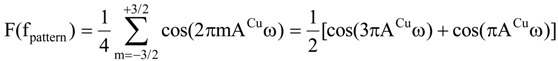
(1)


The second component producing a shift in the field domain can be written in Fourier space:

exp(2πiσω)
(2)

where σ is half of the sweep width of the spectrum.

According to deconvolution theorem and assuming that the nitrogen hyperfine constants are all the same, in agreement with the above assumptions we may write an EPR spectrum in the form:

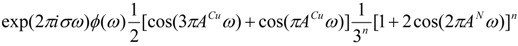
(3)
where *n* is the number of nitrogens and *ϕ*(ω) is a FT-derivative Lorentzian line shape function given by:

ϕ(ω) = −2πiω exp(−2πГω)
(4)

where Г is the half-height width in Gauss.

### 2.2. EPR Spectroscopy of (1:2)Copper(II)-l-Histidine

**Figure 2:**
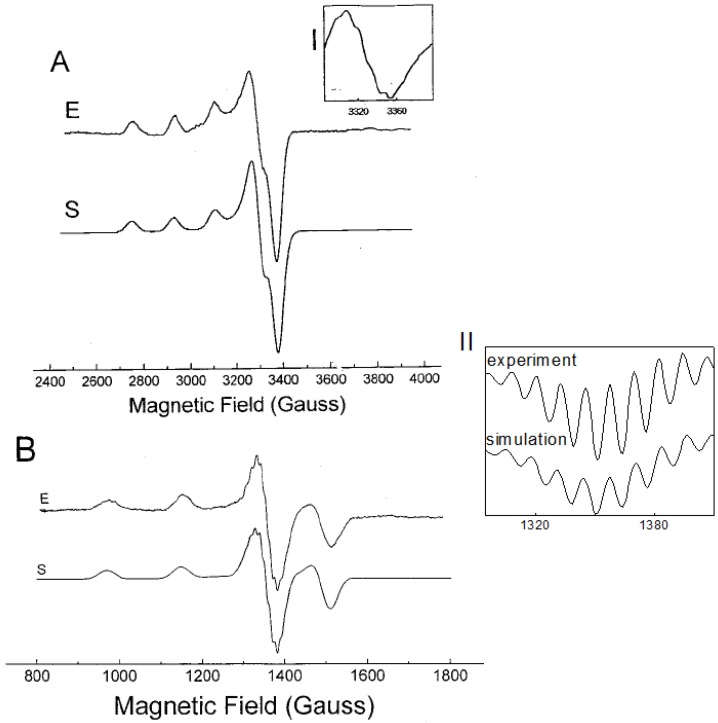
The EPR spectra of [Cu(his)_2_] measured at 77 K and at room temperature. (**A**) spectrum measured at X-band; (**B**) spectrum measured at S-band. Inset I: High-field band of the spectrum measured at X-band at room temperature. Inset II: 2nd derivative of the high field band of the S-band EPR spectrum at 77 K. E-experiment, S-computer simulation (EPR data are given in Table 1).

The EPR spectrum of the (1:2) Cu^2+^-l-His complex at pH 7.3 recorded at X-band at 77 K is presented in Figure 2A. The spectrum is resolved in parallel direction of the g-tensor and shows three of four well-resolved low-field parallel lines with a hyperfine splitting of 554 MHz and *g*_||_ = 2.237. The detailed inspection of the perpendicular band reveals a slight rhombic distortion with *g* values of 2.044 and 2.047. The super-hyperfine splitting due to the directly bonded atoms is not seen in the low-temperature spectrum. Better resolution was observed in the X-band EPR spectrum measured at room temperature, however one should bear in mind that the structure of the copper-histidine complex at low temperature may differ from the structure equilibrated at room temperature. Nevertheless, the X-band EPR spectrum measured at room temperature shows an isotropic quartet, with partially resolved hyperfine structure on the high field line (see Inset I, Figure 2). However, detailed interpretation of the partially resolved multiplet is not straightforward.

In order to obtain better resolution in the superhyperfine splitting due to the directly bonded ligands, S-band EPR spectra at both room temperature and at 77 K were measured (shown in Figure 2B). It is generally known, that the EPR measurements at frequencies lower that 9 GHz (S-band or L-band) can result in: (a) reduced local strain effects, the so called “g-strain” and correlated “g-A” strain; (b) increased resolution of super-hyperfine splitting due to the directly bonded ligand atoms to metal; (c) suppression of spectral line-width and nuclear quadrupole interaction; and finally (d) improved resolution of ligand super-hyperfine splitting due to the enhanced admixture of electronic and nuclear spin functions which in turn increase probability of spectral transitions [22,23,24,25,26].

Generally, l-histidine is one of the most strongly coordinating aminoacids to metal ions possessing three potential metal-binding sites (Figure 1) [27]. They are imidazole imido nitrogen, amino nitrogen and carboxylate oxygen. The dissociation of the second proton of the imidazole group does not occur at physiological pH (pK_a_ = 14). Nitrogen has two stable isotopes, ^14^N (natural abundance 99.632%, spin S = 1) and ^15^N (natural abundance 0.368%, spin S = ½). In the EPR spectra only couplings to nitrogen-14 can be detected. The EPR spectrum measured at low temperature at X-band is shown in Figure 2A. The resolution of the hyperfine splitting structure was further improved by the use of S-band EPR spectroscopy. While the S-band spectrum measured at low temperature (Figure 2B) shows well resolved superhyperfine patterns in the perpendicular region of the spectrum, the EPR spectrum taken at room temperature lacks sufficient resolution (spectrum not shown). The second derivative high-field band of the spectrum (Figure 2B, Inset II), shows nine resolved lines. The best fit to experimental spectrum was achieved assuming the 4N:3NO ratio of 0.8:0.2 with superhyperfine splitting constants summarized in Table 1. The spectral assignment would correspond to a major abundance of metal species formed with both histidines in a histamine-like (4-N) coordination (see below) [28,29], however presence of minor species containing 3-NO coordination mode cannot be excluded (approximately 4:1 ratio in favour of 4-N coordination).

**Table 1:** Parameters used to simulate EPR spectra at S-band for [Cu(his)_2_] ^a,b^.

Complex	g_1_	g_2_	g_3_	A_1_(Cu)	A_2_(Cu)	A_3_(Cu)	A_1_(N)	A_2_(N)	A_3_(N)
[Cu(his)_2_]	2.044	2.047	2.237	27	27	555	38/33 ^c^	38/33 ^c^	38/33 ^c^

^a^ Hyperfine and superhyperfine coupling constants are given in MHz; ^b^ Fluid solution EPR data (X-band and S-band): *g*_iso_ = 2.117, 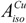 = 199 MHz. Nitrogen shf structure not satisfactorily resolved; ^c^ Simulation of low temperature EPR spectrum was performed using a mixture of four nitrogens (4N, splitting constant = 38 MHz) and three nitrogens (3NO, splitting constant = 33 MHz) assuming the ratio 4N:3NO = 0.8:0.2.

Spin Hamiltonian parameters g_||_ and A_||_ reflect the donor atoms from the ligands. Plots of g_||_ *versus* A_||_ are called Peisach – Blumberg diagrams and suggest the nature of the directly bonded donor atoms to copper ion for cupric complexes [30]. The g_||_ value increases and A_||_ value decreases as oxygen replaces either nitrogen or sulphur atom. A typical g_||_ value is 2.21 and a typical A_||_ value is 570 MHz for 4 nitrogen donor set, while g_||_ of 2.42 and A_||_ of about 390 MHz can be expected for 4 oxygen donor atoms. The spin Hamiltonian parameters for Cu-His (1:2) complex obtained by computer simulation (Table 1) show the g_||_ value of 2.237 and A_||_ value of about 555 MHz. The data are in agreement with either a homogenous 4-N donor set or a mixed 4-N/3-NO donor set with minor abundance of 3-NO structures. 

### 2.3. Structural Characterization of Cu(L-His)_2_ Complex

l-Histidine can bind as mono-, bi- and tri-dentate ligand and its binding mode to a metal depends on the pH of the solution. At physiological pH the major structure of l-histidine is shown in Figure 1. Upon increase of pH the proton from the amino group is deprotonated to give a monoanionic L^−^ ligand. Conversely, upon decrease of pH imidazole nitrogen and carboxylate oxygen are protonated.

Copper(II)-l-histidine species characterized upon increase of pH are: [Cu(HisH)]^2+^ (**MHL**), [Cu(His)]^+^ (**ML**), [Cu(HisH)_2_]^2+^ (**MH_2_L_2_**), [Cu(His)(HisH)]^+^ (**MHL_2_**), [Cu(His)_2_] (**ML_2_**) and [Cu(His)_2_(OH)] (**MH_−1_L_2_**) [5]. The most abundant structures are **MHL**, **MHL_2_** and **ML_2_**. At physiological pH, the predominant structures (more than 99%) in solution are Cu(L-His)_2_ (**ML_2_**).

**Figure 3:**
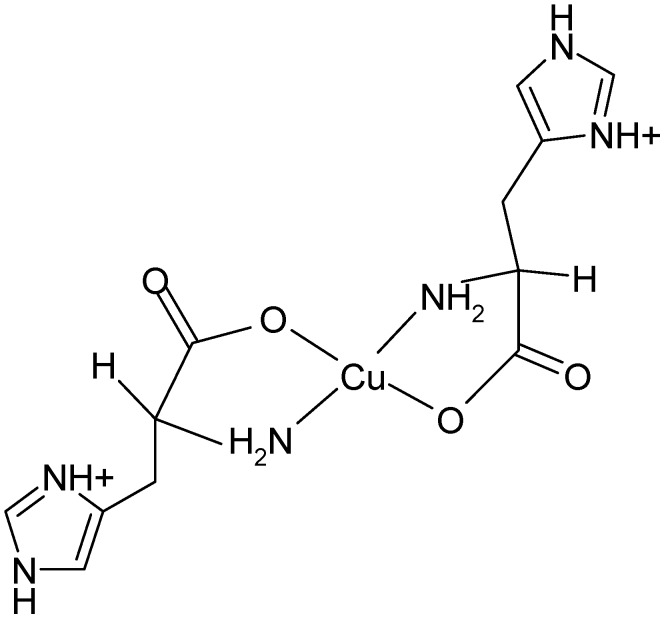
The structure of the Cu(l-His)_2_ complex at acidic pH.

Copper(II) salt mixed with two equiv of l-histidine in aqueous solution results in the pH of approximately 3.7 [5]. The solid state monocrystal X-ray structural analysis of this complex shows four coordinate square-planar arrangement around copper ion with nitrogen and oxygen atoms in trans coordination (Figure 3) [31].

**Figure 4:**
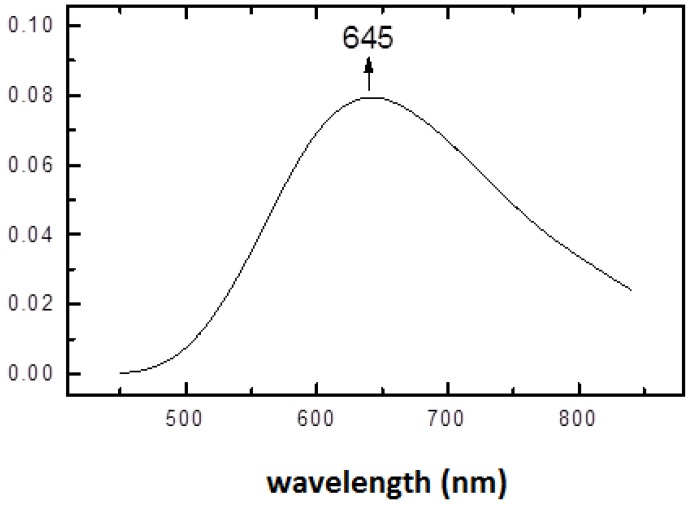
Absorption spectrum of (1:2) copper-histidine complex in PBS at room temperature.

**Figure 5:**
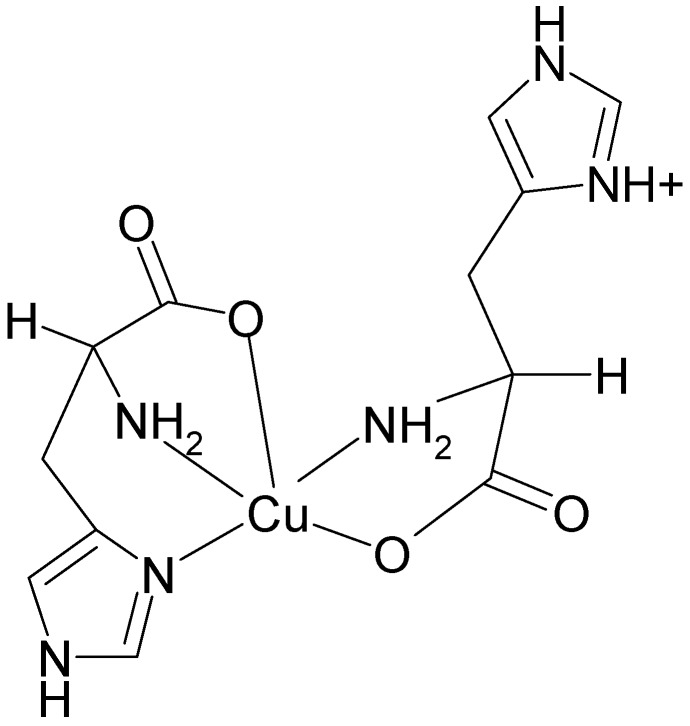
The structure of the Cu(L-His)_2_ complex at physiological pH.

Upon increase of pH to a physiological value (7.2), the structural rearrangement of histidine ligands takes place leading to formation of a square pyramidal copper(II) complex. The electronic spectrum of (1:2) copper-histidine complex in fluid solution is shown in Figure 4. The absorption maximum was observed at relatively long wavelength of 645 nm, in agreement with weak apical chelation by a donor atom of greater ligand field strength than water, as proposed on the basis of ESEEM analysis [32]. Thus, in this complex, copper is coordinated by carboxylate oxygen and amino nitrogen of one histidine molecule and imidazole and amino nitrogens and carboxylate oxygen in apical position of another histidine molecule via semi-coordination (see Figure 5). The imidazole of l-histidine plays an important role in copper chelation, not only in various metalloenzymes but also in thermodynamic stabilization of various copper-amino acid complexes [33]. The tridentate coordination of l-histidine to copper ion provides enhanced stability over binary copper(II) amino acid complexes.

### 2.4. Fourier Transform of the CW-EPR Spectrum of Cu(L-His)_2_

**Figure 6:**
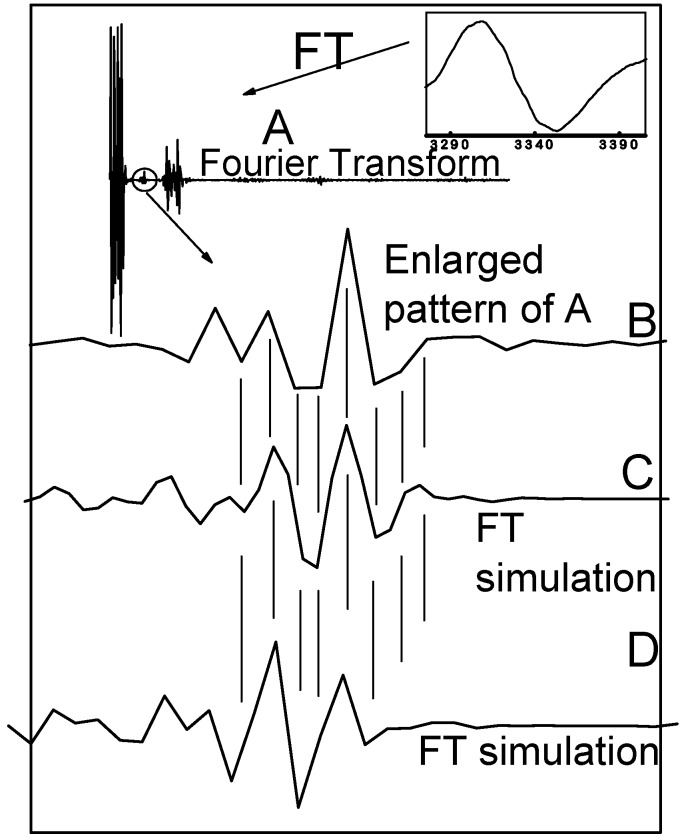
Fourier transform of the X-band CW-EPR spectrum of the complex [Cu(l-His)_2_] ([Cu] = 2 mM) in the phosphate buffer at pH = 7.3. (**A**) Fourier transform of the X-band CW EPR spectrum measured at room temperature. Insets: High-field band of the EPR spectrum measured at room temperature. (**A**) Real part of the FT-EPR spectrum; (**B**) Enlargement of the circled region; (**C**) Part of the simulated FT-EPR spectrum assuming four nitrogens; (**D**) Part of the simulated FT-EPR spectrum assuming three nitrogens.

In addition to detailed interpretation of CW-EPR spectra, Fourier transformation of the CW-EPR spectrum measured at room temperature can be of help to discriminate between even and odd number of directly bonded nitrogens to copper (see Figure 6). It has been observed that fourier transforms consist of a few significant peaks separated by long regions where all the hyperfine components are approaching an extreme value, while the flat regions correspond to those points where one or more of the hyperfine components are small [20,21]. As already observed by other authors, the central region of the fourier transform is very sensitive to the parity of directly bonded magnetically active atoms (nitrogens) [20,21]. The phase of the enlarged region is inverted by varying number of the nitrogens. Fourier transform of the fluid EPR spectrum of Cu(l-His)_2_ measured at room temperature is shown in Figure 6. Inset B shows the phase-sensitive enlarged pattern of the Fourier transform of the fluid EPR spectrum of Cu(l-His)_2_ at pH 7.3. Insets C and D are calculated FT-EPR spectra according to eqn. (3) assuming four and three nitrogens, respectively.

Looking at the match between Fourier transform of the experimental spectrum and simulated FT-EPR spectra using three/four coordinated nitrogens, one can see that the experimental spectrum is a superposition of both simulated spectra with major abundance of species containing four coordinated nitrogens to copper ion and a minor abundance of species with three coordinated nitrogens.

**Figure 7:**
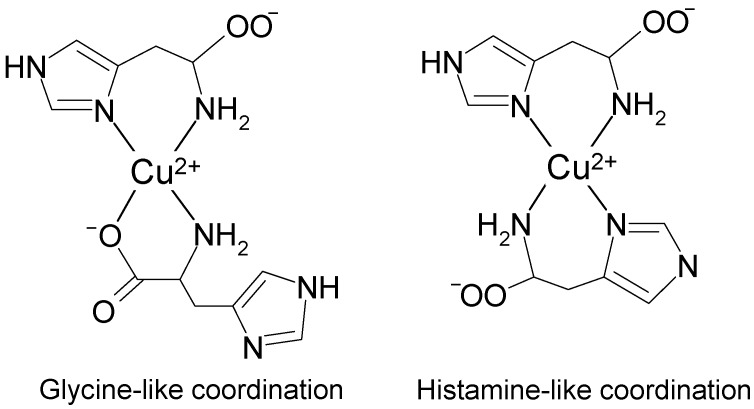
Glycine-like (3-NO) and histamine-like (4-N) types of coordination of two molecules of histidine to copper(II) ion in an aqueous solution at pH 7.3.

Thus in fluid buffer solution at pH 7.3 we can expect an equilibrium between histamine-like (4-N) and glycine-like (3-NO) coordination of two molecules of histidine to copper atom in the [Cu(l-His)_2_] complex (Figure 7).

## 3. Experimental

### 3.1. Metal-Chelate Solutions

L-Histidine and CuCl2∙2H2O were obtained from Sigma Chemical Co. (Milwaukee, WI, USA). Chelate solutions were prepared by mixing the ligand with the metal salt in Hepes buffer (pH 7.3). The concentration of the metal was [Cu] = 2 mM. The metal to chelator ratio was 1:2 throughout the work. The pH was measured using a glass electrode connected to Digi-Sense pH/mV/ORP meter (Cole-Parmer, Vernon Hills, IL, USA).

### 3.2. EPR Measurements

The EPR spectra were recorded on a Bruker EMX spectrometer (X-band) (Bruker BioSpin, Karlsruhe, Germany) coupled to an Aspect 2000 computer and equipped with a variable temperature unit. For low-temperature measurements cylindrical quartz sample tubes with 3.5 mm o.d. (3.0 mm i.d.) were used. The low-temperature EPR spectrum of copper-L-histidine was also recorded at S-band on a Bruker ESP 300 E spectrometer at the National EPR Research Facilities at the University of Manchester, UK. Room temperature measurements at X-band were carried out using a flat cell. In g factor evaluations field gradients were corrected using the internal reference standard marker (g_M_ = 2.0052). The CW-EPR spectra were simulated on an IBM compatible computer PC using the QPOW program [34,35] and a program developed in our laboratory [36].

## 4. Conclusions

In this work we have demonstrated the potential of Fourier-transform CW-EPR spectroscopy in the elucidation of magnetically active atoms directly bonded to metal ions. The results based on the analysis of Peisach-Blumberg plots for spin Hamiltonian parameters and Fourier transform-CW EPR spectroscopy of [Cu(l-His)_2_] complex in water solution at physiological pH revealed an equilibrium between major abundance of histamine-like (4N) and minor abundance of glycine-like (3NO) species.
